# Supplementary figures and images for: SPANXN2 functions a cell migration inhibitor in testicular germ cell tumor cells
Source: PeerJ. 2020 Jun 23;8:e9358. doi: 10.7717/peerj.9358 (PMC7319028; doi:10.7717/peerj.9358)

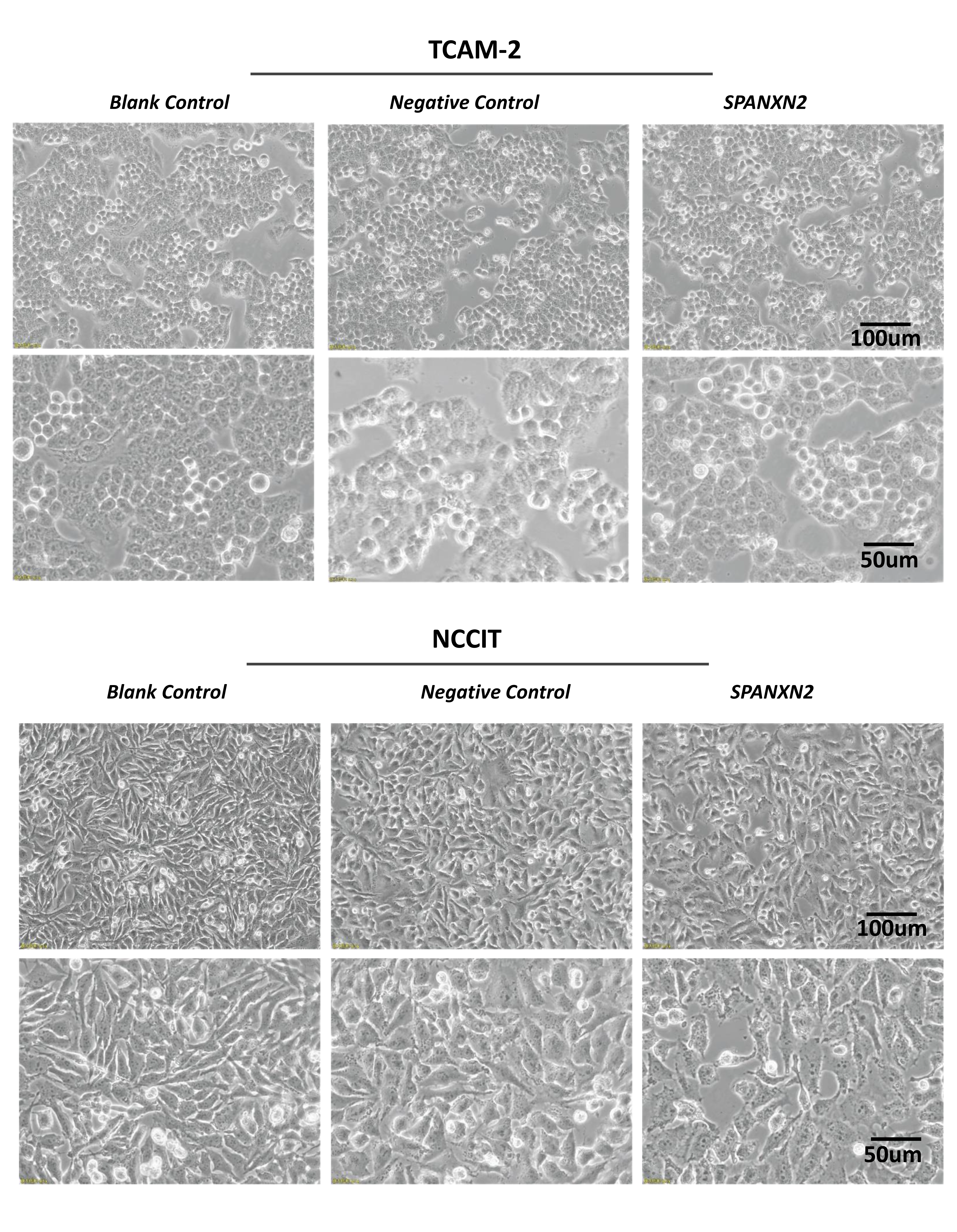

Supplement: Figure S1 — The cell morphology unchanged after SPANXN2 transfected 48h. [file peerj-08-9358-s001.png]

|  | TCAM-2 |         | NCCIT |         |
|--|--------|---------|-------|---------|
|  | NC     | SPANXN2 | NC    | SPANXN2 |

Vimentin

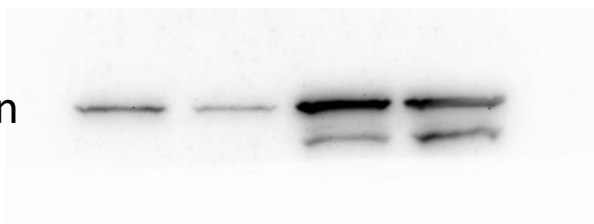

Snail

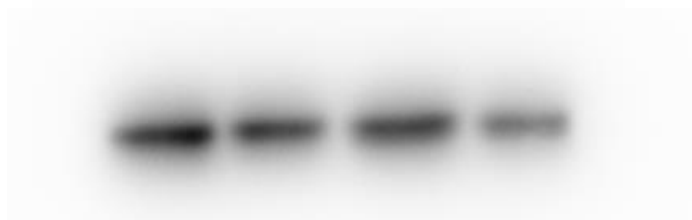

$\alpha$ -Tubulin

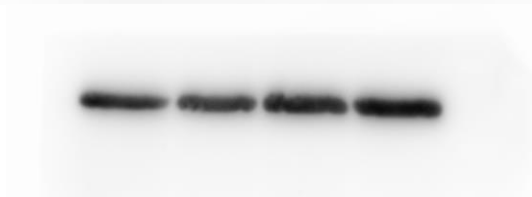

p-AKT

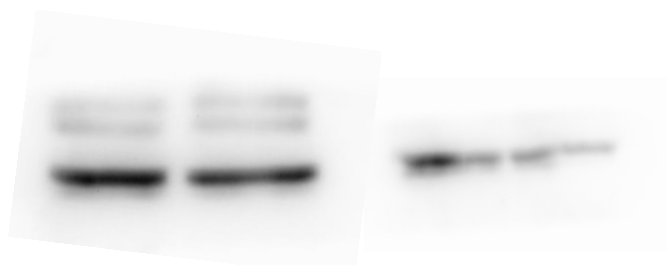

AKT

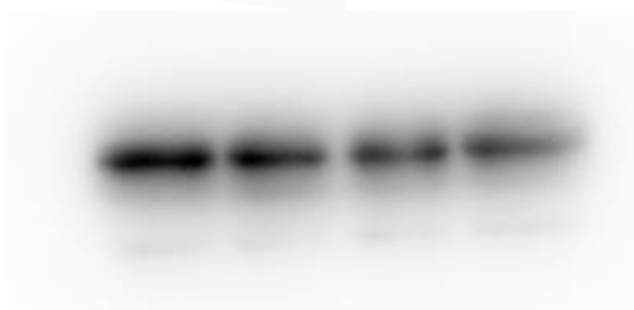

GAPDH

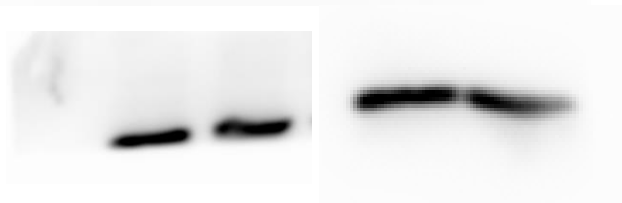

Supplement: File S3 [file peerj-08-9358-s004.pdf]
